# Cutting-edge developments in computer-aided anti-HIV drug design

**DOI:** 10.3389/fimmu.2026.1873962

**Published:** 2026-09-15

**Authors:** Weiying Yang, Ye Wang, Chenda Feng, Wenjing Tang, Lichao Sun

**Affiliations:** 1 Department of Emergency Medicine, First Hospital of Jilin University, Changchun, China; 2 School of Life Sciences, Jilin University, Changchun, China

**Keywords:** acquired immunodeficiency syndrome (AIDS), computer-aided drug design (CADD), deep learning, drug discovery, human immunodeficiency virus (HIV)

## Abstract

Acquired Immunodeficiency Syndrome (AIDS), caused by the Human Immunodeficiency Virus (HIV), remains a major global public health issue. Current antiretroviral therapy (ART) does not fully eradicate HIV infection, and the virus can establish latent reservoirs in the body that are challenging to eliminate. Consequently, the development of new anti-HIV medications remains a critical necessity. Traditional drug development methods are time-consuming, financially demanding, and carry a high risk of failure. In contrast, computer-aided drug design (CADD) offers a promising alternative by leveraging computational techniques and bioinformatics data to accelerate drug development, reduce costs, and improve the success rates. This literature review provides a comprehensive overview of the CADD process, identifies key proteins involved in the HIV life cycle that serve as drug targets. It highlights successful examples where CADD has contributed to the development of inhibitors targeting these proteins. Additionally, the review discusses emerging strategies and limitations of CADD in anti-HIV drug research, offering insights into its potential to revolutionize future therapeutic efforts.

## 1 Introduction

Acquired immunodeficiency syndrome (AIDS), which was first recognized in 1981, is a sexually transmitted infection (STI) and a blood-borne disease affecting humans, currently ranking among the top three infectious diseases globally (1). The primary pathogen responsible for AIDS is human immunodeficiency virus type I (HIV-1) (2, 3). Since the onset of the AIDS epidemic, approximately 42.3 million individuals [ranging from 35.7 million to 51.1 million] have succumbed to AIDS-related conditions, according to World Health Organization (WHO) data (http://UNAIDS.org). The predominant mode of HIV transmission is sexual contact, which includes unsafe homosexual, heterosexual, and bisexual interactions (4, 5). Other transmission routes encompass mother-to-child transmission (which can occur during pregnancy, childbirth, or breastfeeding) and transmission through blood and blood products (such as sharing needles for intravenous drug use, unsafe medical practices, and tattooing) (6, 7). The WHO reports that HIV remains a significant global public health issue, having resulted in 40.4 million deaths to date [with estimates between 32.9 million and 51.3 million] and continues to spread worldwide.

HIV was first isolated and characterized in 1983 and is classified as a single-stranded ribonucleic acid (RNA) lentivirus with a genome comprising nine distinct genes, each transcribed into specific proteins (8–10). Based on genetic and antigenic differences, HIV is divided into two types: HIV-1 and human immunodeficiency virus (HIV-2), with HIV-1 being the more virulent and widely transmitted strain (11–13). The HIV-1 genome is categorized into three major groups, M, N, and O, along with numerous subtypes and variants (14). While HIV-1 is the primary driver of the global pandemic, HIV-2 is mostly restricted to West Africa (15, 16). The viral life cycle of HIV involves several key components, including reverse transcriptase (RT), integrase (IN), protease (PR), glycoprotein 120 (gp120), and glycoprotein 41 (gp41), which serve as potential drug targets for treating HIV infections (17, 18). HIV-1 reverse transcriptase is a crucial enzyme that facilitates the conversion of single-stranded viral RNA (ssRNA) into double-stranded deoxyribonucleic acid (dsDNA), which then integrates into the host’s genomic DNA. This enzyme exhibits both RNA-dependent and DNA-dependent DNA polymerase activity, as well as RNase H activity, playing a vital role in the early stages of the viral life cycle. HIV-1 integrase is responsible for inserting the dsDNA of the viral genome, produced by reverse transcriptase, into the host’s chromosomal DNA. Additionally, HIV-1 protease, a dimeric aspartic acid protease, is tasked with breaking down Gag-Pol polypeptides into viral proteins. The envelope glycoprotein gp120 (Gp120) is found on the surface of the HIV envelope, while gp41 is involved in the fusion of the virus with the host cell.

As of now, the primary approaches for preventing and treating HIV consist of vaccination and antiretroviral therapy (ART). The scientific community largely agrees that the best strategy to manage and eventually eradicate the HIV epidemic is to create a safe, effective, affordable, and easily accessible preventive vaccine for AIDS on a global scale (19, 20). Recent progress in HIV-1 vaccines includes the ability to stimulate the production of broadly neutralizing antibodies (bNAbs) against the HIV-1 virus. Additionally, the HIV-1 envelope glycoprotein (Env) trimer is identified as the protein responsible for starting viral entry and is the exclusive target for broadly neutralizing antibodies (bNAbs) (21). So far, the only Phase III clinical trial for an HIV-1 vaccine that has demonstrated effectiveness is RV144 (22). Nevertheless, the advancement of vaccine development has been significantly impeded by the considerable variability exhibited by HIV-1 reverse transcriptase. The typical duration for the development of conventional vaccines, commencing from the preclinical phase, exceeds a decade (23). It is estimated that the development of a new vaccine candidate utilizing current technologies would incur costs exceeding $500 million, in addition to supplementary expenses ranging from $50 million to $700 million for the establishment of necessary facilities and equipment. HIV exhibits a significant degree of genetic variability, with substantial differences observed among its subtypes, complicating the creation of a vaccine that can effectively address all variant forms of the infection. Furthermore, HIV possesses a remarkable capacity for immune evasion, allowing it to circumvent the host’s immune response. Consequently, no viable HIV vaccine has been developed to date. This underscores the urgent need for the development of effective antiretroviral drugs against HIV. However, the research and development process for new pharmaceuticals is inherently complex, characterized by substantial costs, considerable risks, and extended timelines. The development of an innovative drug typically requires an investment of billions of dollars and a timeframe of 10 to 15 years before it can be brought to market (24).

PR, RT, and IN represent the primary targets for the development of potential anti-AIDS pharmacological agents (25, 26). **Table 1** presents a compilation of pharmaceuticals that have received approval from the U.S. Food and Drug Administration (FDA) for the management of HIV. This includes various classes of antiretroviral agents, such as nucleoside/nucleotide reverse transcriptase inhibitors (NRTIs), non-nucleoside reverse transcriptase inhibitors (NNRTIs), protease inhibitors (PIs), integrase strand transfer inhibitors (INSTIs), fusion inhibitors, C-C chemokine receptor type 5 (CCR5) antagonists, post-cluster of differentiation 4 (CD4) attachment inhibitors, and gp120 attachment inhibitors (27).

**Table 1:** Currently available HIV drugs, target proteins, and indications.

Drug combination programs	Target proteins	Indications	Ref
Nevirapine	RT	HIV infections	(28)
Efavirenz	RT	HIV infections; Alzheimer disease; Prostatic neoplasms	(29)
Ritonavir	PI	HIV infections; Kaposi’s sarcoma;	(30)
Dolutegravir	IN	Breast NeoplasmsNe	(31)
Bictegravir	IN	HIV infections	(32)
Lenacapavir	CA	HIV infections	(33)
Enfuvirtide	(fusion)	HIV infections	(34)
Maraviroc	CCR5	HIV infections; Stroke; Kidney Diseases	(35)
Fostemavir Tromethamine	(attachment)	HIV infections	(36)

The objective of this review is to conduct a systematic examination of the utilization of computer-aided drug design (CADD) in the context of small-molecule drug research for HIV, alongside recent advancements in associated domains. This review will encompass an overview of the evolution of CADD methodologies and tools, an exploration of their applications in HIV drug research, and a comprehensive analysis of the biological characteristics and potential drug targets associated with HIV (**Figure 1**). Furthermore, we will highlight the significant role of CADD in virtual screening, drug design, and toxicity assessment. Additionally, we will address the contributions of CADD in emergency response scenarios during outbreaks and consider prospective future developments in this field.

**Figure 1:**
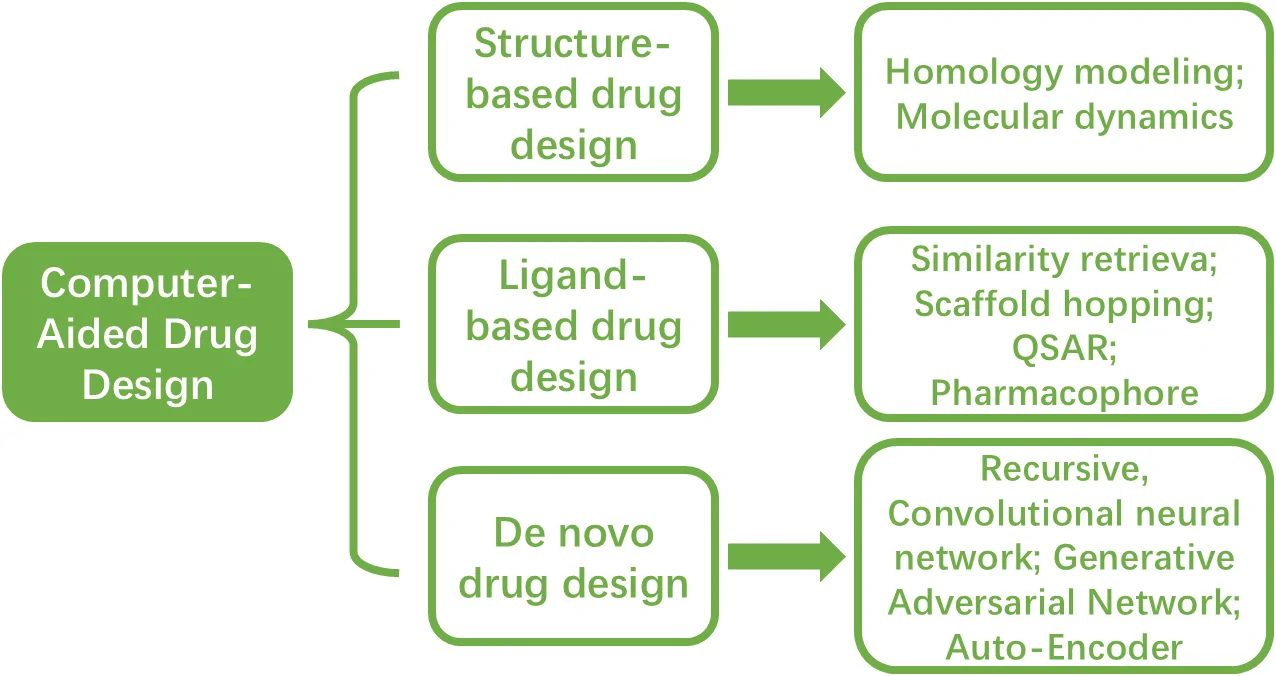
Flow chart of commonly used computer-aided drug design (CADD) methods.

While recent reviews frequently discuss computational anti-HIV drug discovery, they generally concentrate on isolated targets or traditional docking methodologies. The academic community still lacks a systematic evaluation of methodological evolution in CADD. Specifically, current literature misses a comprehensive analysis of the transition from static docking to dynamic simulations, such as molecular dynamics (MD) and Molecular Mechanics/Generalized Born Surface Area (MM/GBSA). Furthermore, we need a clearer understanding of how researchers integrate emerging artificial intelligence-driven *de novo* design across all major HIV enzymes (RT, IN, and PR). Our review stands apart from previous publications because we do more than simply summarize recent active compounds. We critically assess validation deficits in current computational pipelines, notably the omission of absorption, distribution, metabolism, excretion, and toxicity (ADMET) profiling. Ultimately, we provide a standardized methodological blueprint to bridge the gap between in silico predictions and *in vitro*/*in vivo* realities. This work offers researchers actionable insights to develop next-generation therapies against multidrug-resistant HIV strains.

## 2 Research methodology

This manuscript explicitly serves as a narrative review, aimed at systematically summarizing the application value of computer-aided drug design (CADD) in anti-HIV drug research and development. A comprehensive literature search was performed using PubMed and Web of Science. In addition, the official FDA website (www.accessdata.fda.gov) was used to retrieve relevant anti-HIV drug prescribing information. The publication date limits spanned from January 1, 2021, to April 30, 2026. The core search string with Boolean operators was: ((HIV OR AIDS) AND (computer-aided drug design OR CADD) AND (reverse transcriptase OR integrase OR protease OR gp120 OR gp41)). Only English-language publications were included. The inclusion criteria were original research articles, review papers, and official FDA prescribing information relevant to the application of CADD in anti-HIV drug development. Exclusion criteria comprised conference abstracts, preprints, non-English publications, and studies with no direct relevance to CADD techniques or HIV drug targets. Regarding the screening procedure, the initial database search yielded 789 potentially relevant records. After removing 28 duplicate records, titles and abstracts were screened, and approximately 200 records were retained for full-text assessment. During full-text screening, each record was evaluated with respect to study content, target relevance, and the application of CADD techniques. Records with irrelevant themes, redundant information, or failure to meet inclusion criteria were excluded. Finally, 119 eligible records were included in this narrative review.

## 3 HIV life cycle

The HIV life cycle represents a multifaceted biological process encompassing several critical stages: attachment, fusion, and entry into the host cell; reverse transcription of viral RNA; integration; transcription; translation; assembly; and subsequent release of viral progeny (**Figure 2**). Throughout this cycle, various interactions between the virus and the host cell are established. Antiretroviral therapies often target specific proteins that are integral to distinct phases of the viral life cycle. Notably, the HIV envelope glycoprotein, gp120, engages with the CD4 receptor and chemokine co-receptors CCR5 or C-X-C chemokine receptor type 4 (CXCR4) located on the host cell surface. This interaction facilitates the fusion of the viral envelope with the host cell membrane, allowing the virus to penetrate and infect normal cells within the organism (37, 38). The viral genome, along with essential enzymes including RT, IN, and PR, is released into the cytoplasm of the host cell. Subsequently, the HIV viral capsid interacts with the nuclear pore complex (NPC) and facilitates its entry into the nucleus of the cell (39). HIV viral RNA undergoes reverse transcription facilitated by intra-nuclear RT, after which IN incorporates the reverse transcribed viral DNA into the chromosomal DNA of the host cell (37, 40). The viral DNA that is integrated into the host genome is referred to as the provirus, which remains in a latent state until it is triggered by the host cell (41). The protovirus undergoes transcription into messenger RNA (mRNA) facilitated by the host cell’s RNA polymerase II. Subsequently, the mRNA is spliced and transported from the nucleus to the cytoplasm (42). Viral mRNA is translated into viral proteins by the ribosomes of the host cell (43). Gag polyproteins undergo cleavage by viral proteases, resulting in the production of structural proteins such as matrix (MA), capsid (CA), and nucleocapsid (NC) (39, 44). The Gag-Pol polyprotein undergoes cleavage to yield viral enzymes, such as RT, IN, and PR (45). Viral proteins and RNA genomes are organized into viral particles at the membrane of the host cell. Proteins such as Gag and Gag-pol interact with the viral RNA genome to create the nucleocapsid core (46). The core is encased by a lipid bilayer that originates from the membrane of the host cell and incorporates the viral envelope glycoprotein. Ultimately, fully developed viral particles exit the host cell membrane, acquiring the envelope during this process (47–49). This process necessitates the collaboration of viral RNA, Gag-Pol precursors, various enzymes, and membrane proteins.

**Figure 2:**
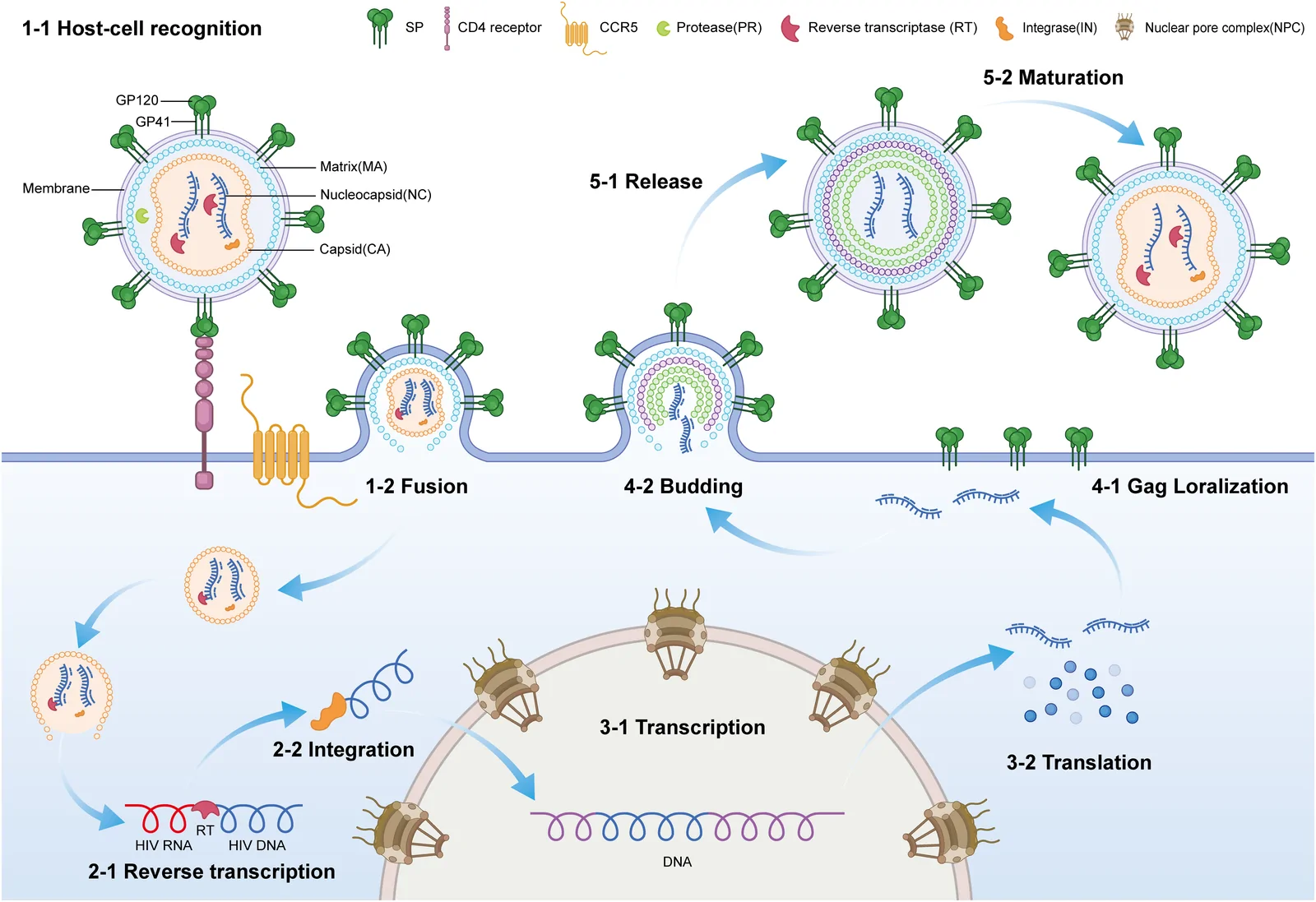
Schematic diagram of the human immunodeficiency virus type 1 (HIV-1) life cycle: Step 1: The virus attaches to the host cell surface via the gp120 protein on the surface of HIV-1, then fuses with and enters the cell; Step 2, viral RNA is reverse transcribed into DNA by the enzyme reverse transcriptase (RT), and the viral DNA is integrated into the host cell's genome by integrase (IN); Step 3, the integrated viral DNA, or provirus, is transcribed into RNA and translated into viral proteins by the host cell's machinery; Step 4, the newly synthesized viral proteins and RNAs self-assemble into new viral particles at the host cell membrane, and the viral protease (PR) cleaves the Gag and Gag-Pol polyproteins into their functional forms; Step 5, once the viral particles are assembled, they bud from the host cell and are released, ready to infect additional cells.

## 4 Application of computer-aided drug design

CADD employs computational and bioinformatics techniques to facilitate drug discovery and development, significantly reducing research expenditures compared to conventional methodologies (50). The prevalent generic CADD approaches encompass structure-based drug design (SBDD), ligand-based drug design (LBDD), and *de novo* drug design (DNDD). Key techniques employed in anti-HIV drug discovery include MD simulation (51), molecular docking (52), MM/GBSA (53), virtual screening (VS) (54), pharmacological modeling (55), quantitative structure-activity relationship (QSAR) modelling (56), and assessments of ADMET (57). These tools, used independently or in combination with experimental methods, have facilitated inhibitor repurposing, novel drug design, and binding affinity optimization (58).

For targets with known three-dimensional structures, SBDD leverages molecular docking and virtual screening informed by experimental or predicted structures from databases such as the Protein Data Bank (PDB), with AlphaFold and RosettaFold providing valuable structural predictions when experimental data are unavailable (59–61). Using repositories like PubChem (62), researchers can efficiently screen or design small-molecule ligands, construct complex structures through molecular simulations, and rigorously evaluate binding energies and physicochemical properties (63).

LBDD is a strategy employed in the identification of lead compounds through the analysis of the structural characteristics of ligands that exhibit specific binding affinity to a target, particularly in instances where the three-dimensional structure of the protein target remains undetermined (64). Widely utilized methods in ligand-based drug design (LBDD) encompass similarity searches, scaffold hopping, QSAR, and pharmacophore modeling (65).

Furthermore, recent advancements in artificial intelligence (AI) and machine learning (ML) have markedly progressed across various scientific fields. The convergence of these technologies with computer-aided drug design is transforming the drug discovery process. *De novo* drug design represents a significant domain that has experienced substantial advancements due to generative AI and diffusion models (66). Unlike traditional generative adversarial networks, diffusion models excel at generating 3D molecular structures by learning the complex probability distributions of molecular geometries, enabling the direct generation of novel, synthesizable anti-HIV scaffolds that are stereochemically stable. To further refine these generated structures, reinforcement learning (RL) algorithms are increasingly integrated with generative frameworks. By employing multi-objective reward functions, RL dynamically optimizes the molecules, simultaneously maximizing critical parameters such as target binding affinity, quantitative estimate of drug-likeness (QED), and synthetic accessibility (SA) to ensure the clinical viability of theoretical candidates (67). Additionally, graph neural networks (GNNs) have significantly improved the extraction of molecular features by capturing complex inter-atomic topological connections, outperforming traditional 1D and 2D molecular representations in predicting drug-target binding affinities (68). Models powered by artificial intelligence, such as target-conditioned transformer models, can now utilize AlphaFold-derived latent protein embeddings to seamlessly generate target-specific molecules across diverse protein families (68). Furthermore, multi-step high-throughput virtual screening pipelines integrate graph neural networks with absorption, distribution, metabolism, and excretion (ADME) predictions and drug-likeness rules to ensure optimal pharmacokinetic profiles (68).

## 5 Function and structure of target proteins for anti-HIV drug candidates

### 5.1 Reverse transcriptase

RT is a critical enzyme in the HIV infection lifecycle, serving two primary functions: it acts as an RNA-dependent DNA polymerase (RDDP) and a DNA-dependent DNA polymerase (DDDP) (**Figure 3**). Additionally, it possesses ribonuclease H (RNase H) activity, which enables it to degrade the RNA component of RNA/DNA double-stranded hybrids (69). The initiation of host cell infection involves the transcription of the viral single-stranded RNA (ssRNA) genome into double-stranded DNA (dsDNA) facilitated by the enzyme reverse transcriptase. Consequently, reverse transcriptase is regarded as a critical molecular target in the development of therapeutic agents aimed at combating HIV (70).

**Figure 3:**
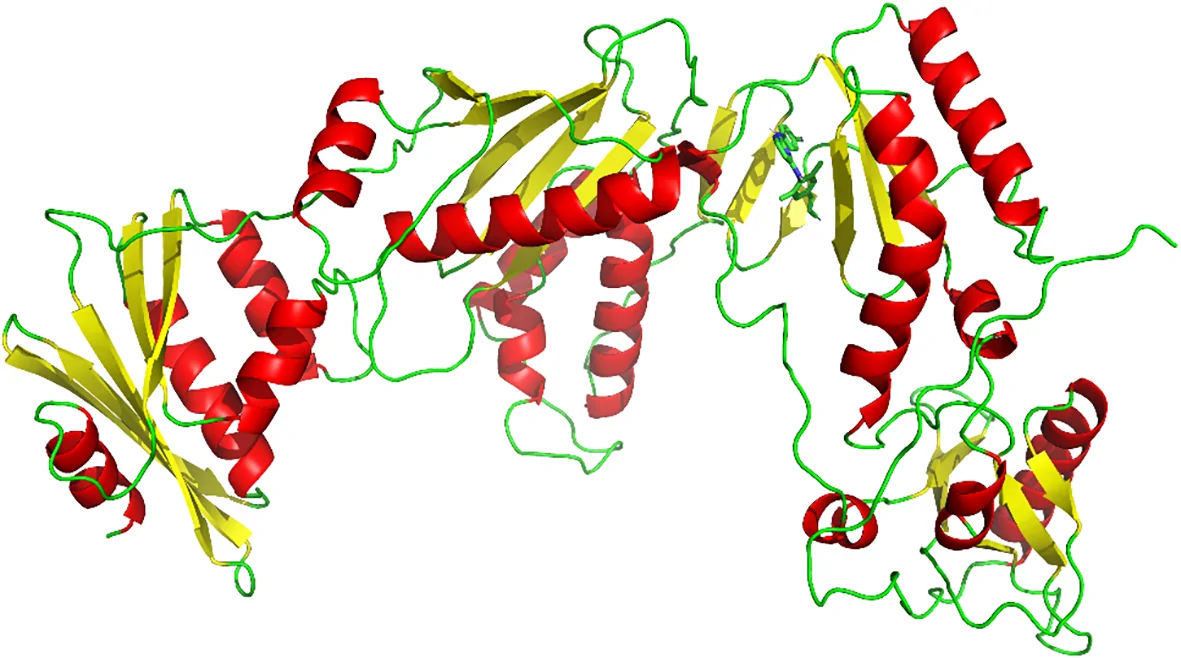
Structure of HIV-1 reverse transcriptase (RT) in complex with the non-nucleoside reverse transcriptase inhibitor (NNRTI) rilpivirine (PDB ID: 4G1Q). HIV-1 RT is a heterodimer illustrating a complex architecture composed of a p66 subunit and a p51 subunit. The structural domains of the subunit—finger, palm, thumb, and connection-are represented in the form of red and yellow cartoons. The NNRTI Rilpivirine is indicated by yellow cartoon, bound within the active site of the enzyme. The key residues in the inhibitor binding site are indicated by green sticks, highlighting their interaction with the inhibitor. The protein structure was displayed and analyzed using the PyMOL visualization program, providing a detailed view of the enzyme-inhibitor interaction and the overall architecture of the complex.

The structural organization of HIV-1 RT, including its subunit architecture, functional domains, and key residues within the NNRTI binding pocket, is illustrated in **Figure 3**. HIV reverse transcriptase (RT) is a heterodimer with a molecular weight of 117 kDa, composed of a 66 kDa subunit (p66) and a 51 kDa subunit (p51), which is derived from p66. The p66/p51 HIV-1 reverse transcriptase possesses a functional DNA polymerase activity as well as an active site for ribonuclease H (RNase H), both of which are situated in spatially distinct regions within the p66 subunit (71). The structural domain of the p66 polymerase within reverse transcriptase (RT) is categorized into four distinct sub-structural domains: the finger domain, comprising residues 1–84 and 119-154; the palm domain, encompassing residues 85–118 and 155-241; the thumb domain, which includes residues 242-313; and the linkage domain, consisting of residues 314-426 (72). The p51 subunit is derived from the proteolytic cleavage of p66, which does not possess the structural domain characteristic of RNase H (73). As illustrated in **Figure 3**, the inhibitor binds tightly within the active site. Specifically, key amino acid residues, including Lys101, Tyr181, Tyr188, and Trp229, form critical interactions within the binding pocket, determining the spatial conformation and therapeutic efficacy of the compound against the HIV-1 RT target. Antiretroviral medications primarily consist of NRTIs and NNRTIs. NRTIs function by competitively inhibiting the HIV reverse transcriptase enzyme, thereby obstructing the reverse transcription process of viral RNA into DNA (48, 74). Frequently utilized nucleoside reverse transcriptase inhibitors (NRTIs) encompass zidovudine (AZT) (75), lamivudine (3TC) (76), and abacavir (ABC) (77), among others. NNRTIs function by directly binding to the inactive domain of reverse transcriptase, resulting in a conformational alteration that inhibits its enzymatic activity. Notable examples of NNRTIs include nevirapine (NVP) (78), efavirenz (EFV) (29), and rilpivirine (RPV) (79). Nevirapine interacts with HIV-1 reverse transcriptase, inhibiting its catalytic site and consequently obstructing the replication of the virus (2, 80). Three frequently utilized non-nucleoside reverse transcriptase inhibitors (NNRTIs), namely efavirenz, nevirapine, and delavirdine, demonstrate efficacy in suppressing the replication of wild-type (WT) HIV strains. However, their effectiveness is significantly diminished against clinically relevant RT mutant strains, such as Y188C, Y181C, K103N, and L100I. These mutant viruses have acquired substantial resistance to the majority of currently available NNRTIs (81).

To address the challenges posed by clinically relevant reverse transcriptase mutant strains, such as K103N, researchers are increasingly turning to graph neural networks (GNNs) enhanced by self-supervised learning (SSL) pre-training strategies (68). By leveraging robust node-level and graph-level representations of molecular structures, computational screening can efficiently navigate data scarcity to rapidly identify novel non-nucleoside reverse transcriptase inhibitors (NNRTIs) that bypass traditional resistance mutations (68). Furthermore, these ML-based predictions are successfully integrated with in-silico structure-based screening, including molecular docking and MD simulations, continuously refining repurposed candidate molecules to maximize binding affinity against mutant strains while preserving favorable pharmacokinetic profiles (68). A comprehensive comparison of the computational methodologies, validation strategies, and limitations across different HIV-1 targets (RT, IN, and PR) discussed in this section is summarized in **Table 2**.

**Table 2:** This table provides a comparative overview of computational methodologies applied to the discovery and optimization of inhibitors targeting HIV-1 reverse transcriptase (RT), integrase (IN), and protease (PR).

Target	Study	Computational methodology	Software/tools	Validation strategy	Representative compounds	Biological activity	Advantages	Limitations
RT	Sakakibara et al. (82)	Docking, bioisostere strategy	MacroModel (AMBER* force field)	>3,000 ligand conformers generated	22 triazine-based NNRTIs	4c: EC_50_: 0.068μM 5: EC_50_: 0.11μM	Identified key H-bonds and π–π stacking	Rigid receptor; lacks MD simulations
RT	Su et al. (83)	Conformational analysis, minimization	MMFFs force field	X-ray crystallography	Tetrahydroquinoline A1	IC_50_: 1.1nM (WT)	High crystallographic reliability	No receptor flexibility or free-energy calculations
RT	Zhang et al. (84)	Rigid docking	AutoDock Vina	Superimposition with active compounds	S-DABO compounds	6c1: EC_50_: 0.24μM	Mapped critical H-bond networks	Empirical scoring only; cytotoxicity issues
RT	Chander et al. (85)	Cross-docking, ADMET prediction	Schrödinger (Glide XP), QikProp	Redocking (RMSD 0.6–1.1 Å)	Piperazine derivative 6b	EC_50_: 4.72μg/ml	Insights into multi-mutant resistance	Neglects water-mediated polar interactions
IN	Patel et al. (96)	Docking-based design	Not specified	Correlation with antiviral assays	Tricyclic pyrimidinone carboxamides	3: WT EC_50_: 1nM	Structural rationale for SAR	Lacks redocking validation and MD
IN	Sharma et al. (97)	Docking, homology modeling	Not specified	Modeled full-length IN with DNA	Phenanthrene β-diketo acids	18: ST IC_50_: 1.3μM	Highly biologically relevant model	No explicit protein flexibility or ADMET
IN	Oliaie et al. (102)	Molecular docking	AutoDock Vina	Superposition with raltegravir	2-hydroxyacetophenones	7: EC_50_: 40–45μM 9: EC_50_: 45μM	Replicated reference binding mechanism	No MM/GBSA or in silico ADMET
PR	Wang et al. (103)	MB-QSAR, Partial Least Squares	SYBYL 6.9	Quantified steric/electrostatic contributions	Comprehensive PR dataset	Correlation coefficients r 2 pred> 0.7	Strong external predictive performance	Cannot capture mutation-induced flap dynamics
PR	Zhou et al. (111)	Docking, homology modeling	Schrödinger suite	FRET techniques	Pyrrolidine 34b	IC_50_: 0.32 nM, EC_50_: 1.90 μM (MDR)	Efficacy against MDR variants	Static models without dynamic validation

Key methodological parameters, including the specific software tools, validation strategies, and the biological activities of representative compounds (IC_50_ and EC_50_ values), are summarized. Additionally, the table highlights the methodological advantages and critical limitations of each study, particularly regarding the integration of molecular dynamics (MD) simulations, receptor flexibility considerations, and in silico ADMET (absorption, distribution, metabolism, excretion, and toxicity) predictions.

RT, reverse transcriptase; IN, integrase; PR, protease; NNRTI, non-nucleoside reverse transcriptase inhibitor; WT, wild-type; MDR, multidrug-resistant; MD, molecular dynamics; ADMET, absorption, distribution, metabolism, excretion, and toxicity; RMSD, root mean square deviation; FRET, fluorescence resonance energy transfer; MB-QSAR, mutation-dependent biomacromolecular quantitative structure-activity relationship.

To explore key interactions within the non-nucleoside inhibitor binding pocket (NNIBP), Sakakibara and colleagues employed a classical bioisostere strategy to design and synthesize 22 novel triazine-based NNRTIs, and conducted molecular docking studies using MacroModel software with the AMBER* force field (82). Their workflow generated over 3,000 ligand conformers and successfully identified key hydrogen bonds (e.g., between the 6-amino group and the Lys101 backbone) and hydrophobic contacts, including π-π stacking with Tyr181 and Tyr188 (82). However, this study exemplifies the limitations of the early Docking-only paradigm: its scoring function was not validated by MD simulations to assess complex stability, nor were binding free energies estimated using methods such as Molecular Mechanics/Poisson–Boltzmann Surface Area (MM/PBSA) or MM/GBSA. Moreover, the receptor was treated as rigid, completely neglecting protein flexibility. As a result, while the predicted binding modes could rationalize activity, they lacked dynamic validation and reliability.

Methodologically more advanced is the work by Su and co-workers. In their investigation of tetrahydroquinoline-based allosteric inhibitors, they integrated computational modeling with X-ray crystallography. Their workflow generated approximately 100 ligand conformations and employed energy minimization with the MMFFs force field, successfully predicting that a cis-cyclopropyl ring could serve as a structural surrogate for a sulfur-methylene linker, a hypothesis subsequently validated by experimental potency (the cis-enantiomer 16) and crystallographic structures (PDB: 3I0R and 3I0S) (83). Compared to pure docking approaches, this study demonstrates the higher reliability of a docking and crystallographic validation strategy. Nevertheless, its methodological limitations remain evident. The modeling still focused primarily on ligand conformational analysis without integrating receptor flexibility; MD simulations were not performed to assess the temporal stability of the complexes; and no free-energy calculations were employed to quantitatively rank binding affinities across the series.

Zhang and colleagues utilized AutoDock Vina for rigid docking of S-DABO compounds based on the 1rt1 crystal structure. Their study successfully mapped the hydrogen-bond network with key residues Lys101 and Lys103 and, through superimposition with the active compound 4j, qualitatively elucidated how C-6 substituents affect π-π stacking interactions. However, their computational workflow represents another widely adopted static docking paradigm: relying solely on empirical scoring functions to evaluate binding modes, without employing MD simulations to account for target flexibility or performing subsequent free-energy calculations. Consequently, the quantitative interpretation and validation of structure–activity relationship (SAR) trends remain severely constrained (84).

To address clinically relevant drug-resistant mutants, Chander and colleagues employed a ligand-based molecular hybridization strategy to design tetrahydroquinoline-piperazine derivatives (the specific chemical structures of compounds 6a-o are detailed in the original publication) (85). Their computational approach demonstrates significant progress at the level of docking and multi-mutant processing. Using the Glide module (XP mode) within the Schrödinger suite, they performed systematic cross-docking against wild-type (PDB: 3MEE) and two mutant structures (K103N: 3TAM; K103N/Y181C: 4I2Q), and validated the reproducibility of their protocol by redocking co-crystallized ligands (RMSD values of 0.6, 1.1, and 0.9 Å, respectively). More importantly, their analysis successfully correlated the disruption of a dual hydrogen-bond network (ligand with Lys101/Lys103) with mutation-induced structural disorientation, providing computational insights into resistance mechanisms. Furthermore, the integration of ADMET predictions via three platforms (QikProp, admetSAR, and FAF-Drugs) reflects a forward-looking awareness of early-stage drug-likeness filtering. Nevertheless, the depth of their simulations remains limited. The absence of MD simulations and MM/GBSA free-energy calculations to refine binding conformations, and the removal of crystallographic water molecules prior to docking, neglect receptor flexibility and water-mediated polar interactions, thereby limiting the precise quantitative evaluation of relative binding affinities.

Across the above CADD studies targeting RT (**Table 2**), a notable common limitation is the fragmentation of the computational validation pipeline. Across the above CADD studies targeting RT, a notable common limitation is the fragmentation of the computational validation pipeline. Most studies stop at static docking or conformational searches, lacking MD simulation-supported dynamic conformational analysis and binding free-energy calculations (MM/GBSA), which means the predicted binding modes lack thermodynamic and kinetic cross-validation. Even more striking is that virtually none of the studies systematically performed pre-synthetic ADMET filtering (neither Su (83) nor Zhang (84) employed computational ADMET tools; only Su (83) provided experimental PK data post-synthetically). This directly contributes to the precipitous drop in cellular activity observed for some highly potent molecules [such as the cytotoxicity issues noted by Zhang et al. (86)]. This series of methodological discontinuities not only undermines the practical value of computational predictions in guiding synthetic optimization but also highlights the urgent need to establish a standardized docking, MD, MM/GBSA, and ADMET prediction validation and screening pipeline for future RT-targeted CADD research, in order to genuinely bridge the gap between computation and experimentation.

### 5.2 Integrase

HIV-1 IN plays a crucial role in the integration of viral DNA, which is generated through RT, into the host genome (**Figure 4**). This process is vital for the completion of the viral replication cycle, making integrase an indispensable enzyme in the life cycle of HIV-1 (87). INSTIs demonstrate efficacy in obstructing this mechanism. Frequently utilized INSTIs encompass polyenes, including dolutegravir (DTG) (88) and bictegravir (BIC) (89). In contrast to PR and RT, there are currently no identified functional analogs of IN, nor is there substantial evidence of cytotoxic effects associated with IN inhibitors in human cells (90).

**Figure 4:**
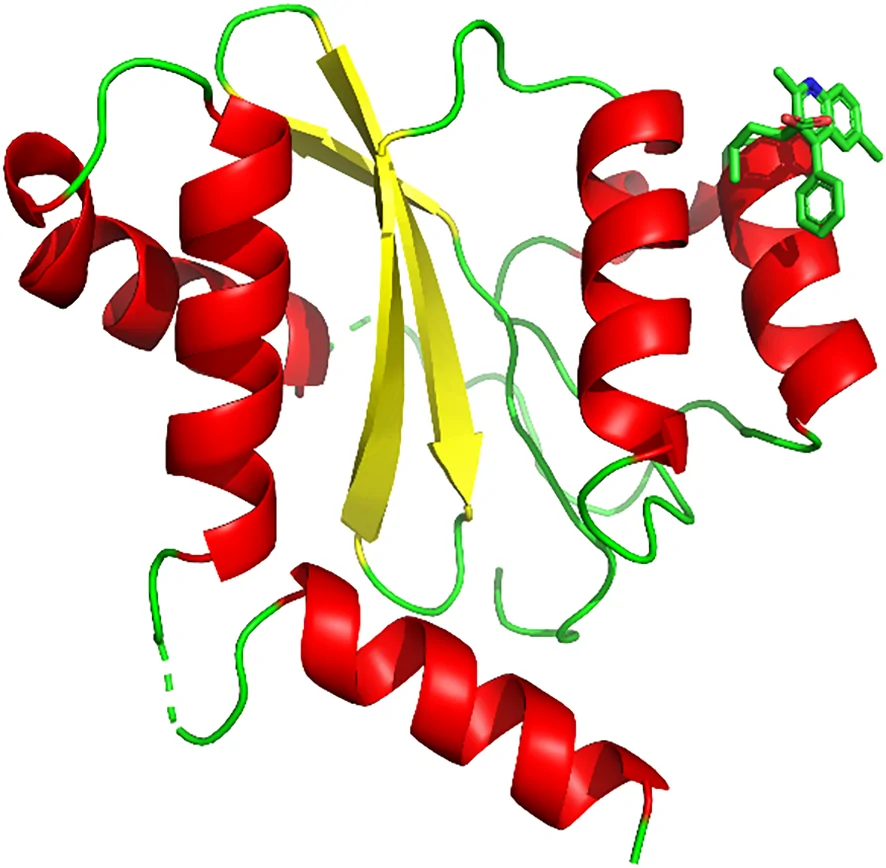
Structure of HIV-1 integrase (IN) in complex with an integrase strand transfer inhibitor (INSTI) (PDB ID: 3LPU). In the dimeric structure, the enzyme's architecture is represented in the form of red and yellow cartoons, which can be categorized into three distinct structural domains: the N-terminal domain (NTD), the catalytic core domain (CCD), and the C-terminal domain (CTD). The key residues in the inhibitor binding site are indicated by green sticks, highlighting their interaction with the inhibitor. The protein structure was displayed and analyzed using the PyMOL visualization program, providing a detailed view of the enzyme-inhibitor interaction and the overall architecture of the complex.

HIV-1 IN is encoded by the pol gene and comprises 288 amino acids, resulting in a molecular weight of 32 kDa. This protein can be categorized into three distinct structural domains: the N-terminal domain (NTD), which spans residues 1 to 49; the catalytic core domain (CCD), encompassing residues 50 to 212; and the C-terminal domain (CTD), which includes residues 213 to 288 (91). The zinc finger located within the N-terminal domain (NTD) contributes to the stability of the integrase enzyme. Additionally, the effective chelation of the DDE motifs—specifically Asp64, Asp116, and Glu152—with the two magnesium ions (Mg^2+^) present in the central catalytic domain (CCD) is essential for sustaining optimal enzymatic activity. Furthermore, the C-terminal domain (CTD) functions as a nonspecific binding site for viral DNA (92, 93).

The structural modeling of HIV-1 integrase, particularly the functional intasome complex, has historically been challenging due to its high flexibility and the lack of complete crystallographic data. The advent of AlphaFold-enabled structure prediction has revolutionized this aspect of integrase inhibitor design (94). AlphaFold and its subsequent iterations allow for the highly accurate prediction of full-length integrase structures and their dynamic conformational states. By utilizing these AI-predicted models, structure-based drug design can now more reliably simulate the binding of novel integrase strand transfer inhibitors within the catalytic core domain and predict how the enzyme interacts with viral DNA, significantly reducing the reliance on static and often incomplete experimental models.

A total of ten primary inhibitors of HIV-1 IN have been identified in the literature. These include diketo acids, diazonaphthalene derivatives, quinolones, pyrimidinones, thiazathiazepines, polyhydroxyaromatic compounds, disulfides, benzenesulfonamides, coumarin derivatives, and salicylhydrazide derivatives, among others (95, 96). Diketo acids represent the most extensively researched and promising inhibitors of HIV-1 IN, exhibiting significant potency, selectivity, and minimal toxicity (97). Raltegravir was the inaugural integrase inhibitor to receive approval from the FDA in 2007 (98), subsequently followed by the approvals of second-generation agents with higher resistance barriers such as dolutegravir (99) and bictegravir (100), and more recently, the long-acting injectable formulation cabotegravir (101). As detailed in **Table 2**, several computational pipelines have been employed in recent years to optimize the design of IN inhibitors. Methodologically, Sharma and colleagues advanced beyond earlier Docking-only studies by employing a homology model of full-length HIV-1 IN bound to DNA, constructed from the PFV crystal structure-a clear improvement over their previous work using partial IN structures without DNA (97). Using this more biologically relevant model, the docking successfully identified a novel binding region that did not interact with resistance-associated residues Gln148 and Asn155. However, the docking protocol relied on a conventional scoring function without explicitly treating protein flexibility, and no MD simulations were performed to assess complex stability or refine binding poses. The absence of MM/PBSA or MM/GBSA free-energy calculations further limits the quantitative reliability of the predicted binding modes, representing a notable gap between computational prediction and rigorous validation.

Patel and colleagues employed a docking-based strategy using the PFV intasome structure (PDB: 3OYL) as the template to design bridged tricyclic pyrimidinone carboxamides as IN strand transfer inhibitors (96). Their docking models provided a clear structural rationale for the observed SAR, particularly highlighting the oxamide moiety’s role in hydrogen bonding with the 3’-OH of the terminal adenosine of viral DNA. The predictive utility of the docking received partial validation through correlation with measured antiviral activity. However, like Sharma et al., this study did not perform MD simulations to assess the dynamic stability of the complexes or binding free-energy calculations. Furthermore, the absence of rigorous redocking validation, such as RMSD quantification against known co-crystal structures, represents a notable methodological gap that limits confidence in the predicted binding modes.

Employing a similar docking-only approach, Oliaie and colleagues used AutoDock Vina with the PFV intasome structure (PDB: 3OYA) to design 2-hydroxyacetophenone analogues as IN inhibitors (102). A notable feature of their workflow was the superposition of compound 7’s docked pose with the co-crystallized ligand raltegravir, which demonstrated that the designed compound replicates the reference binding mechanism. The docking results aligned with biological activity data, as the most potent compounds (7 and 9) exhibited the expected chelation interactions with Mg^2+^ ions. However, this study also lacked MD simulations to account for receptor flexibility, MM/PBSA or MM/GBSA free-energy calculations, and redocking validation with RMSD quantification, all of which are essential for establishing the reliability of docking predictions and quantifying the confidence in binding mode hypotheses.

Collectively, these studies demonstrate a common pattern in computational IN inhibitor research: the frequent reliance on docking alone without subsequent dynamic refinement. The absence of MD simulations and free-energy calculations represents a significant limitation, as IN is a flexible enzyme, particularly in the CCD and CTD regions. The lack of rigorous validation protocols, such as redocking co-crystallized ligands to assess RMSD, also undermines confidence in the predicted binding modes. Furthermore, the treatment of receptor flexibility (or lack thereof) is a critical factor that distinguishes more reliable from less reliable computational workflows.

ADMET considerations: an assessment of specific studies. The integration of ADMET prediction into IN inhibitor discovery has been limited but is gaining attention. Among the studies reviewed, none of the above-mentioned IN inhibitor studies (96, 97, 102) performed systematic ADMET profiling of their synthesized compounds. S.S. Oliaie et al. (102) reported experimental cytotoxicity data (CC_50_ values) but did not provide in silico ADMET predictions or discuss drug-likeness parameters. Similarly, Sharma et al. (97) and Patel et al. (96) focused primarily on enzyme inhibition and antiviral activity without assessing pharmacokinetic properties such as absorption, metabolism, or toxicity. This represents a missed opportunity, as early ADMET prediction could have identified potential liabilities such as poor oral bioavailability, high plasma protein binding, or cytochrome P450 inhibition. For example, the limited antiviral activity observed for some compounds in cellular assays may partially reflect poor cell permeability or extensive protein binding, but without systematic ADMET profiling we cannot confirm this. Future studies should incorporate ADMET prediction tools such as QikProp, admetSAR, or SwissADME to provide a more comprehensive assessment of therapeutic potential, as recommended by current CADD best practices (102).

### 5.3 Protease

HIV-1 PR is a critical enzyme in the HIV life cycle, as it facilitates the cleavage of Gag and Gag-Pol polyproteins, resulting in the generation of a singular mature protein (**Figure 5**) (103, 104). The inhibition of HIV PR plays a crucial role in obstructing the maturation and subsequent release of new viral particles, which in turn hinders the dissemination of the virus. Frequently utilized PIs encompass lopinavir (LPV) (86), ritonavir (RTV), among others (105).

**Figure 5:**
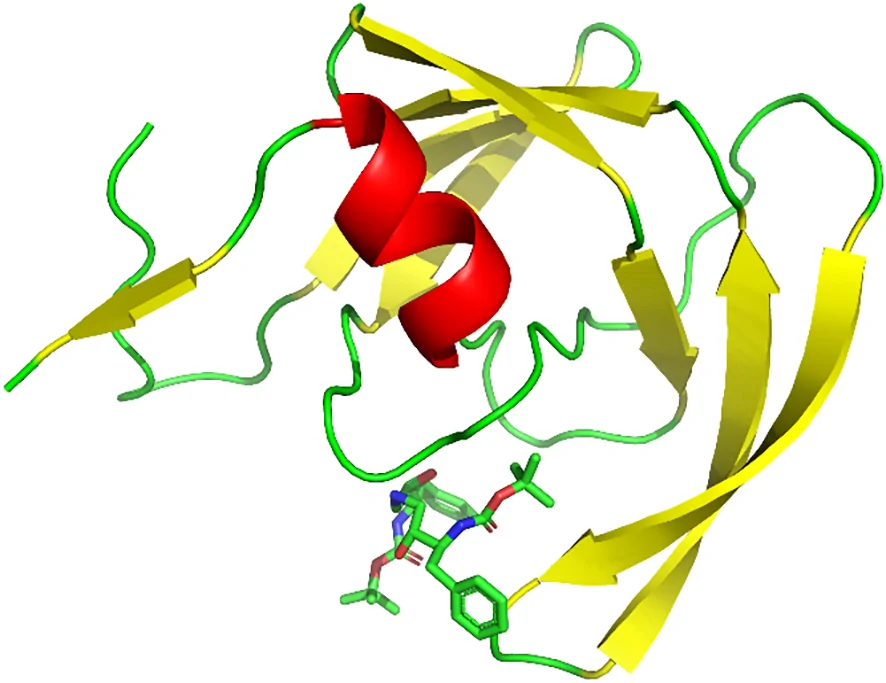
Structure of HIV-1 protease (PR) in complex with a protease inhibitor (PDB ID: 1ODW). HIV-1 PR is a homodimeric aspartic protease, with its structural domains represented in a cartoon model colored by secondary structure (red α-helices, yellow β-sheets, and green loops). The displayed inhibitor is bound within the active substrate-binding pocket, specifically occupying the S1 and S2 subsites, and is shown as sticks. The essential catalytic residues, including the catalytic triad (Asp25, Thr26, and Gly27), are located within this pocket. The flexible flap regions covering the active site are also functionally integral to the complex. The structure was visualized and analyzed through the PyMOL program.

HIV-1 PR is characterized as a homodimeric aspartic acid protease, featuring a substrate-binding pocket that comprises the catalytic triad formed by Asp25(25’), Thr26(26’), and Gly27(27’), along with a valve region (106). The active site of HIV-1 PR is organized into eight distinct subsites, designated as S4, S3, S2, S1, S1’, S2’, S3’, and S4’. These subsites correspond to eight substrate residues labeled as P4, P3, P2, P1, P1’, P2’, P3’, and P4’, with the cleavage bond situated between the residues P1 and P1’ (107). HIV-1 PR has emerged as a significant molecular target for the development of anti-HIV pharmacological agents. The HIV-1 PR domain consists of 99 amino acids and adopts a predominantly β-sheet structure, with each monomer containing one α-helix and two antiparallel β-sheets. The PR functions as a homodimer, and the active site is formed at the dimer interface and covered by flexible β-hairpin (flap) regions. The catalytic residues Asp25 and Asp125 are located within the active-site region, while the flap regions play an important role in substrate and inhibitor recognition and contribute to the formation of the inhibitor-binding pocket (**Figure 6**) (108). This domain corresponds to database entries such as Pfam PF00077 and PROSITE PS00141. Common resistance-associated mutations include V82A, I84V, and L90M, which are situated near the active site or the dimerization interface. These mutations can alter the geometric conformation of the subsites, thereby synergistically reducing inhibitor binding affinity, and represent a critical mechanistic basis for clinical multidrug resistance (MDR) (109).

**Figure 6:**
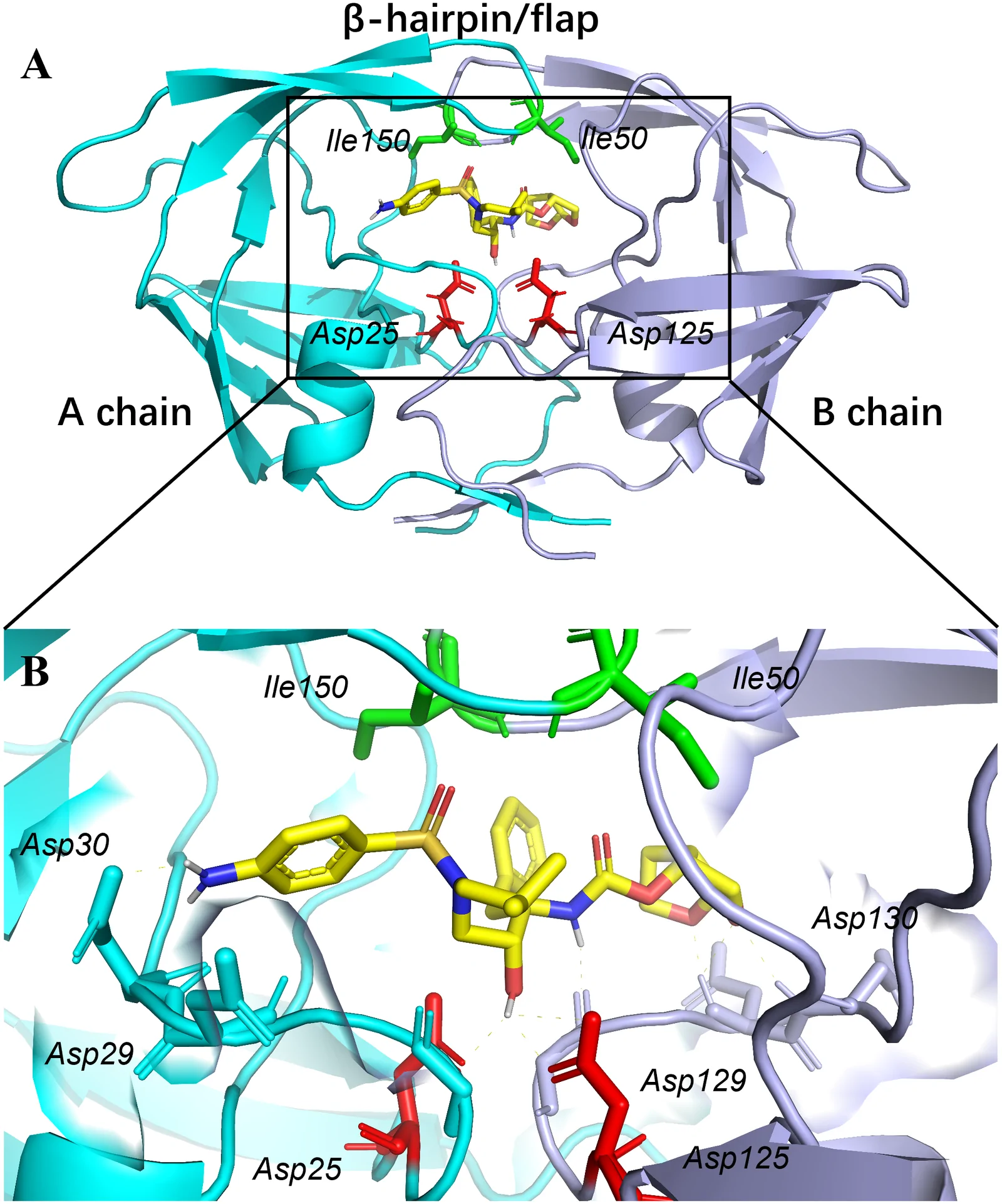
Structural organization and inhibitor-binding site of HIV-1 protease. **(A)** Overall structure of the HIV-1 protease homodimer in complex with darunavir (PDB ID: 2IEN). The A and B chains are shown as cyan and light blue cartoons, respectively, with the catalytic residues Asp25/Asp125 and flap residues Ile50/Ile150 shown as red and green sticks, respectively. **(B)** Close-up view of the darunavir-binding pocket showing representative active-site residues, including Asp25/ Asp125, Asp29/Asp129, Asp30/Asp130, and Ile50/Ile150. Darunavir is shown in yellow stick representation, and hydrogen-bond interactions between darunavir and surrounding residues are indicated by yellow dashed lines.

In the context of HIV-1 protease, predicting how specific mutations confer multidrug resistance remains a significant hurdle. Protein language models (PLMs) have emerged as a cutting-edge solution to this problem (110). By training on vast databases of protein sequences, protein language models acquire rich representational capabilities and can capture the evolutionary constraints and mutational landscapes of the protease enzyme (110). These models can accurately predict the impact of distal minor mutations on the flap dynamics and the overall geometry of the active site (110). Consequently, generative AI methodologies can use these insights to design next-generation protease inhibitors that maintain their efficacy across a broad spectrum of clinical isolates by targeting evolutionarily conserved, mutation-resistant pockets.

To combat these resistance profiles, recent research has heavily relied on CADD, with the methodological characteristics of representative PR-targeted studies outlined in **Table 2**. A representative example of docking-based virtual screening applied to PR is the work of Zhou and colleagues, who designed pyrrolidine-derived P2 ligands and employed the Schrödinger suite for molecular docking and homology modeling against both WT and drug-resistant PR variants (111). Their most potent compound 34b exhibited exceptional enzyme inhibitory activity with an IC_50_ of 0.32 nM and maintained efficacy against multidrug-resistant variants with an EC_50_ of 1.90 µM. The docking models revealed extensive hydrogen bonding and salt bridge networks between the inhibitor and backbone residues of the protease. However, this study relied entirely on static docking models. The authors did not perform MD simulations to assess the stability of the protein-ligand complexes over time, nor did they compute binding free energies using MM/PBSA or MM/GBSA methods. Without these dynamic validation steps, the predicted binding modes lack rigorous confirmation, and the influence of receptor flexibility on inhibitor binding remains uncharacterized.

Recognizing the limitations of static docking for PR, Venkatachalam and colleagues emphasized in their review the critical importance of MD simulations and free-energy calculations for capturing the conformational dynamics of PR mutants (106). Their analysis demonstrates that computational workflows which explicitly account for receptor flexibility, including two-layered ONIOM quantum mechanics-molecular mechanics models, achieve substantially higher predictive reliability for resistance mechanism assessment than rigid docking alone. The review highlights that MD simulations can sample the open and closed conformations of the flaps, providing thermodynamic and kinetic information that docking methods cannot access. This methodological perspective directly addresses the deficiencies observed in docking-only studies and establishes a benchmark for what constitutes a rigorous computational validation pipeline.

As an alternative to computationally expensive MD simulations, Wang and colleagues employed a Mutation-dependent Biomacromolecular Quantitative Structure Activity Relationship (MB-QSAR) method to predict drug resistance across 59 HIV-1 PR mutants (103). Using Comparative Molecular Field Analysis and Comparative Molecular Similarity Indices Analysis with Partial Least Squares regression, they quantified the relative contributions of steric and electrostatic fields to resistance. Steric fields accounted for approximately 60 percent of the resistance-conferring effect, while electrostatic fields contributed roughly 40 percent. The principal advantage of this approach is its strong external predictive performance, with cross-validated correlation coefficients exceeding 0.7 for most inhibitors, combined with rapid computation that avoids the high cost of MD simulations. However, the method depends on pre-aligned static homology models that do not account for the extreme flap dynamics induced by distal minor mutations. This limitation means that while MB-QSAR can efficiently identify resistance-associated structural features, it cannot fully capture the conformational entropy changes that govern inhibitor binding in highly flexible systems.

Consistent with the crucial role of ADMET in CADD, evaluating the pharmacokinetic and toxicological profiles of PR inhibitors is vital for predicting their therapeutic potential. In the aforementioned study by Zhou et al., while *in vitro* cytotoxicity was evaluated (demonstrating a favorable CC_50_>200µM), the computational workflow lacked systematic in silico ADMET predictions. Information regarding absorption, metabolism, membrane permeability, or drug-likeness parameters was not reported, leaving a significant gap in predicting *in vivo* bioavailability and potential drug-drug interactions. Future computational workflows for HIV-1 PR inhibitors must routinely incorporate ADMET prediction tools (e.g., QikProp, admetSAR, or SwissADME) to provide a more holistic assessment of new scaffolds, bridging the gap between high *in vitro* potency and clinical viability.

## 6 Future prospects and conclusions

AIDS, caused by HIV, continues to pose significant challenges for global healthcare. While ART remains the cornerstone of treatment by effectively suppressing viral replication and improving patient outcomes, the virus’s ability to develop resistance underscores the need for innovative drug discovery approaches.

Recently, multi-targeting and multivalent binding strategies have gained prominence in the design of anti-HIV drugs. These innovative approaches aim to enhance therapeutic efficacy and mitigate the emergence of drug resistance by concurrently targeting multiple critical stages or proteins within the HIV life cycle (112, 113). For example, some research teams are developing drugs that can inhibit both HIV RT and IN or designing multivalent inhibitors that bind to several different sites on the HIV surface protein (25, 114).

To achieve these complex polypharmacological goals, the field is transitioning from conventional docking and ligand-based screening to advanced, AI-driven methodologies. Traditional models have already enhanced the precision of QSAR analyses (115–117) and automated high-throughput screening (118). Building on this, cutting-edge technologies-such as GNNs, generative AI, diffusion models, AlphaFold-enabled structure predictions, and transformer-based PLMs-are now shaping the future. These advancements not only drive *de novo* drug design and uncover cryptic binding sites, but also allow for accurate drug-drug interaction predictions and the identification of synergistic effects from combination therapies (119).

The broader application of AI in CADD is currently limited by inconsistent data quality, opaque model interpretability, and high computational demands. Furthermore, predictive models frequently suffer from overfitting. A more fundamental issue is the persistent gap between in silico predictions and experimental reality, which underscores the risk of relying purely on virtual screening metrics without rigorous *in vitro* or *in vivo* validation.

To advance from algorithmic development to clinical utility, the field must adopt stricter validation workflows prior to chemical synthesis. Routine integration of molecular docking, MD simulations, MM/GBSA calculations, and ADMET profiling is necessary to confirm the structural stability and pharmacokinetic viability of generated compounds. Beyond validation, addressing the data deficit is critical. Expanding training databases to include comprehensive structural data on HIV mutant strains will directly improve the generalizability of these models against resistant variants. As these data pipelines mature, generative AI can more effectively accelerate the design of targeted combination therapies, tackling multiple viral targets (e.g., RT, IN, and PR) without compromising overall oral bioavailability.

In conclusion, realizing the full potential of AI in anti-HIV drug discovery requires moving beyond theoretical predictions. By prioritizing robust analytical validation, curating high-quality mutant databases, and focusing on rational combination strategies, researchers can position AI-driven CADD as a reliable foundation for developing next-generation treatments against resistant strains.

## References

[1] Menéndez-Arias L Delgado R . Update and latest advances in antiretroviral therapy. Trends Pharmacol Sci. (2022) 43:16–29. doi: 10.1016/j.tips.2021.10.004 34742581

[2] Battini L Bollini M . Challenges and approaches in the discovery of human immunodeficiency virus type-1 non-nucleoside reverse transcriptase inhibitors. Med Res Rev. (2019) 39:1235–73. doi: 10.1002/med.21544 30417402

[3] Khan S Attique Z Hamza M Li LY Imran M Yu L . A next-generation strategy for HIV-1 vaccination: computational design of a multi-epitope subunit vaccine targeting global circulating variants. Front Virol. (2026) 5:1705632. doi: 10.3389/fviro.2025.1705632

[4] Ng'eno BN Kellogg TA Kim AA Mwangi A Mwangi M Wamicwe J . Modes of HIV transmission among adolescents and young adults aged 10-24 years in Kenya. Int J STD AIDS. (2018) 29:800–5. doi: 10.1177/0956462418758115 29490572 PMC5995643

[5] Del Rio C . The global HIV epidemic: What the pathologist needs to know. Semin Diagn Pathol. (2017) 34:314–7. doi: 10.1053/j.semdp.2017.05.001 28566241 PMC5531065

[6] Kassa GM . Mother-to-child transmission of HIV infection and its associated factors in Ethiopia: a systematic review and meta-analysis. BMC Infect Dis. (2018) 18:216. doi: 10.1186/s12879-018-3126-5 29747581 PMC5946547

[7] Osório D Munyangaju I Nacarapa E Muhiwa A Nhangave AV Ramos JM . Mother-to-child transmission of HIV infection and its associated factors in the district of Bilene, Gaza Province-Mozambique. PloS One. (2021) 16:e0260941. doi: 10.1371/journal.pone.0260941 34890430 PMC8664209

[8] Gallo RC Montagnier L . The chronology of AIDS research. Nature. (1987) 326:435–6. doi: 10.1038/326435a0 3550473

[9] Barré-Sinoussi F Chermann JC Rey F Nugeyre MT Chamaret S Gruest J . Isolation of a T-lymphotropic retrovirus from a patient at risk for acquired immune deficiency syndrome (AIDS). Science. (1983) 220:868–71. doi: 10.1126/science.6189183 6189183

[10] Khan N Chen X Geiger JD . Role of divalent cations in HIV-1 replication and pathogenicity. Viruses. (2020) 12:471. doi: 10.3390/v12040471 32326317 PMC7232465

[11] Visseaux B Le Hingrat Q Damond F Charpentier C Descamps D . Physiopathology of HIV-2 infection. Virologie (Montrouge). (2019) 23:277–91. doi: 10.1684/vir.2019.0789 31826849

[12] Kalinichenko S Komkov D Mazurov D . HIV-1 and HTLV-1 transmission modes: mechanisms and importance for virus spread. Viruses. (2022) 14:152. doi: 10.3390/v14010152 35062355 PMC8779814

[13] Günthard HF Kusejko K Kouyos RD . Phylogenetics and molecular evolution to understand and curb the HIV pandemic. Nat Rev Microbiol. (2026) 24:76–92. doi: 10.1038/s41579-025-01202-w 40588586

[14] Weber IT Wang YF Harrison RW . HIV protease: historical perspective and current research. Viruses. (2021) 13:839. doi: 10.3390/v13050839 34066370 PMC8148205

[15] Visseaux B Damond F Matheron S Descamps D Charpentier C . Hiv-2 molecular epidemiology. Infect Genet Evol. (2016) 46:233–40. doi: 10.1016/j.meegid.2016.08.010 27530215

[16] Boswell MT Rowland-Jones SL . Delayed disease progression in HIV-2: the importance of TRIM5α and the retroviral capsid. Clin Exp Immunol. (2019) 196:305–17. doi: 10.1111/cei.13280 30773620 PMC6514420

[17] Mallakuntla MK Togre NS Santos DB Tiwari S . Implications of fragment-based drug discovery in tuberculosis and HIV. Pharm (Basel). (2022) 15:1415. doi: 10.3390/ph15111415 36422545 PMC9692459

[18] Alexiev I Dimitrova R . The origins and genetic diversity of HIV-1: evolutionary insights and global health perspectives. Int J Mol Sci. (2025) 26:10909. doi: 10.3390/ijms262210909 41303393 PMC12653013

[19] Chen Z Xu J Jin X Wang J Huang J Zhang H . Grand challenges on HIV/AIDS in China - the 5th symposium, Yunnan 2024. Emerg Microbes Infect. (2025) 14:2492208. doi: 10.1080/22221751.2025.2492208 40202047 PMC12016243

[20] Fang Z Jiang W Liu P Xia N Li S Gu Y . Targeting HIV-1 immune escape mechanisms: Key advances and challenges in HIV-1 vaccine design. Microbiol Res. (2025) 299:128229. doi: 10.1016/j.micres.2025.128229 40460810

[21] Leggat DJ Cohen KW Willis JR Fulp WJ deCamp AC Kalyuzhniy O . Vaccination induces HIV broadly neutralizing antibody precursors in humans. Science. (2022) 378:eadd6502. doi: 10.1126/science.add6502 36454825 PMC11103259

[22] Rerks-Ngarm S Pitisuttithum P Nitayaphan S Kaewkungwal J Chiu J Paris R . Vaccination with ALVAC and AIDSVAX to prevent HIV-1 infection in Thailand. N Engl J Med. (2009) 361:2209–20. doi: 10.1056/NEJMoa0908492 19843557

[23] Pronker ES Weenen TC Commandeur H Claassen EH Osterhaus AD . Risk in vaccine research and development quantified. PloS One. (2013) 8:e57755. doi: 10.1371/journal.pone.0057755 23526951 PMC3603987

[24] DiMasi JA Grabowski HG Hansen RW . Innovation in the pharmaceutical industry: New estimates of R&D costs. J Health Econ. (2016) 47:20–33. doi: 10.1016/j.jhealeco.2016.01.012 26928437

[25] Yang F Yang J Zhang Z Tu G Yao X Xue W . Recent advances in computer-aided antiviral drug design targeting HIV-1 integrase and reverse transcriptase associated ribonuclease H. Curr Med Chem. (2022) 29:1664–76. doi: 10.2174/0929867328666210708090123 34238145

[26] Zhang C Xie Q Wan CC Jin Z Hu C . Recent advances in small-molecule HIV-1 integrase inhibitors. Curr Med Chem. (2021) 28:4910–34. doi: 10.2174/0929867328666210114124744 33459224

[27] Labh R Gupta R . Emerging trends in the long-acting antiretroviral therapy: current status and therapeutic challenges. Curr HIV Res. (2021) 19:4–13. doi: 10.2174/1570162x18666200824104140 32838720

[28] Ha B Larsen KP Zhang J Fu Z Montabana E Jackson LN . High-resolution view of HIV-1 reverse transcriptase initiation complexes and inhibition by NNRTI drugs. Nat Commun. (2021) 12:2500. doi: 10.1038/s41467-021-22628-9 33947853 PMC8096811

[29] Costa B Vale N . Efavirenz: history, development and future. Biomolecules. (2022) 13:88. doi: 10.3390/biom13010088 36671473 PMC9855767

[30] Hui L Xiaoxu H Yuqi W Peng W Xin W Yunyun Y . Effectiveness and safety analysis of PIs/r based dual therapy in treatment-naïve, HIV/AIDS patients: A network meta analysis of randomized controlled trials. Front Pharmacol. (2022) 13:811357. doi: 10.3389/fphar.2022.811357 35308227 PMC8931831

[31] Scarsi KK Havens JP Podany AT Avedissian SN Fletcher CV . HIV-1 integrase inhibitors: A comparative review of efficacy and safety. Drugs. (2020) 80:1649–76. doi: 10.1007/s40265-020-01379-9 32860583 PMC7572875

[32] Mbhele N Chimukangara B Gordon M . HIV-1 integrase strand transfer inhibitors: a review of current drugs, recent advances and drug resistance. Int J Antimicrob Agents. (2021) 57:106343. doi: 10.1016/j.ijantimicag.2021.106343 33852932

[33] Segal-Maurer S DeJesus E Stellbrink HJ Castagna A Richmond GJ Sinclair GI . Capsid inhibition with lenacapavir in multidrug-resistant HIV-1 infection. N Engl J Med. (2022) 386:1793–803. doi: 10.1056/NEJMoa2115542 35544387

[34] Su X Wang Q Wen Y Jiang S Lu L . Protein- and peptide-based virus inactivators: inactivating viruses before their entry into cells. Front Microbiol. (2020) 11:1063. doi: 10.3389/fmicb.2020.01063 32523582 PMC7261908

[35] Xiao T Cai Y Chen B . HIV-1 entry and membrane fusion inhibitors. Viruses. (2021) 13:735. doi: 10.3390/v13050735 33922579 PMC8146413

[36] Markham A . Fostemsavir: first approval. Drugs. (2020) 80:1485–90. doi: 10.1007/s40265-020-01386-w 32852743

[37] van Heuvel Y Schatz S Rosengarten JF Stitz J . Infectious RNA: human immunodeficiency virus (HIV) biology, therapeutic intervention, and the quest for a vaccine. Toxins (Basel). (2022) 14:138. doi: 10.3390/toxins14020138 35202165 PMC8876946

[38] Elalouf A Elalouf H Maoz H . HIV-1 entry mechanisms: protein-host receptor interactions and membrane fusion dynamics. Front Biosci (Landmark Ed). (2025) 30:37412. doi: 10.31083/fbl37412 41198533

[39] Masenga SK Mweene BC Luwaya E Muchaili L Chona M Kirabo A . HIV-host cell interactions. Cells. (2023) 12:1351. doi: 10.3390/cells12101351 37408185 PMC10216808

[40] Cabrera-Rodríguez R Pérez-Yanes S Lorenzo-Sánchez I Trujillo-González R Estévez-Herrera J García-Luis J . HIV infection: shaping the complex, dynamic, and interconnected network of the cytoskeleton. Int J Mol Sci. (2023) 24:13104. doi: 10.3390/ijms241713104 37685911 PMC10487602

[41] Elliott JL Kutluay SB . Going beyond integration: the emerging role of HIV-1 integrase in virion morphogenesis. Viruses. (2020) 12:1005. doi: 10.3390/v12091005 32916894 PMC7551943

[42] Murray JM Kelleher AD Cooper DA . Timing of the components of the HIV life cycle in productively infected CD4+ T cells in a population of HIV-infected individuals. J Virol. (2011) 85:10798–805. doi: 10.1128/jvi.05095-11 21835801 PMC3187481

[43] Lerner G Weaver N Anokhin B Spearman P . Advances in HIV-1 assembly. Viruses. (2022) 14:478. doi: 10.3390/v14030478 35336885 PMC8952333

[44] Sever B Otsuka M Fujita M Ciftci H . A review of FDA-approved anti-HIV-1 drugs, anti-gag compounds, and potential strategies for HIV-1 eradication. Int J Mol Sci. (2024) 25:3659. doi: 10.3390/ijms25073659 38612471 PMC11012182

[45] Available online at: https://bio.libretexts.org/Bookshelves/Microbiology/Microbiology_(Kaiser)/Unit_4%3A_Eukaryotic_Microorganisms_and_Viruses/10%3A_Viruses/10.06%3A_Animal_Virus_Life_Cycles%3A_An_Overview/10.6A%3A_The_Productive_Life_Cycle_of_Animal_Viruses (Accessed May 1, 2026).

[46] Shcherbatova O Grebennikov D Sazonov I Meyerhans A Bocharov G . Modeling of the HIV-1 life cycle in productively infected cells to predict novel therapeutic targets. Pathogens. (2020) 9:255. doi: 10.3390/pathogens9040255 32244421 PMC7238236

[47] Kleinpeter AB Freed EO . HIV-1 maturation: lessons learned from inhibitors. Viruses. (2020) 12:940. doi: 10.3390/v12090940 32858867 PMC7552077

[48] Tompa DR Immanuel A Srikanth S Kadhirvel S . Trends and strategies to combat viral infections: A review on FDA approved antiviral drugs. Int J Biol Macromol. (2021) 172:524–41. doi: 10.1016/j.ijbiomac.2021.01.076 33454328 PMC8055758

[49] Gupta M Pak AJ Voth GA . Critical mechanistic features of HIV-1 viral capsid assembly. Sci Adv. (2023) 9:eadd7434. doi: 10.1126/sciadv.add7434 36608139 PMC9821859

[50] Rizzi A Mandelli D . High performance-oriented computer aided drug design approaches in the exascale era. Expert Opin Drug Discov. (2025) 20:1–10. doi: 10.1080/17460441.2025.2468289 39953911

[51] Yan D Ma Y Chen X Deng S Wang Q . Molecular dynamics-driven drug discovery. Phys Chem Chem Phys. (2025) 27:12633–51. doi: 10.1039/d5cp00380f 40488609

[52] Ansari RM Mundke RN Agrawal YO Goyal SN Nakhate KT Rathod SS . Recent advances in molecular docking techniques: Transforming perspectives in distinct drug targeting and drug discovery approaches. Med Chem. (2025) 22:30. doi: 10.2174/0115734064384472250716144736 40696555

[53] Pan YL Liu YL Chen JZ . Computational simulation studies on the binding selectivity of 1-(1H-benzimidazol-5-yl)-5-aminopyrazoles in complexes with FGFR1 and FGFR4. Molecules. (2018) 23:767. doi: 10.3390/molecules23040767 29584670 PMC6017917

[54] da Rocha MN de Sousa DS da Silva Mendes FR Dos Santos HS Marinho GS Marinho MM . Ligand and structure-based virtual screening approaches in drug discovery: Minireview. Mol Divers. (2025) 29:2799–809. doi: 10.1007/s11030-024-10979-6 39223358

[55] Kaserer T Beck KR Akram M Odermatt A Schuster D . Pharmacophore models and pharmacophore-based virtual screening: Concepts and applications exemplified on hydroxysteroid dehydrogenases. Molecules. (2015) 20:22799–832. doi: 10.3390/molecules201219880 26703541 PMC6332202

[56] Wang T Yuan XS Wu MB Lin JP Yang LR . The advancement of multidimensional QSAR for novel drug discovery - Where are we headed? Expert Opin Drug Discov. (2017) 12:769–84. doi: 10.1080/17460441.2017.1336157 28562095

[57] Kar S Leszczynski J . Open access in silico tools to predict the ADMET profiling of drug candidates. Expert Opin Drug Discov. (2020) 15:1473–87. doi: 10.1080/17460441.2020.1798926 32735147

[58] Bonardi A . Computational approaches for designing viral protease inhibitors. Enzymes. (2025) 58:59–91. doi: 10.1016/bs.enz.2025.06.005 41238303

[59] Finn J . Application of SBDD to the discovery of new antibacterial drugs. Methods Mol Biol. (2012) 841:291–319. doi: 10.1007/978-1-61779-520-6_13 22222458

[60] Li L Liu S Wang B Liu F Xu S Li P . An updated review on developing small molecule kinase inhibitors using computer-aided drug design approaches. Int J Mol Sci. (2023) 24:13953. doi: 10.3390/ijms241813953 37762253 PMC10530957

[61] Fatima Ali N Khan S Zahid S . A critical address to advancements and challenges in computational strategies for structural prediction of protein in recent past. Comput Biol Chem. (2025) 117:108430. doi: 10.1016/j.compbiolchem.2025.108430 40121710

[62] Kim S Thiessen PA Bolton EE Chen J Fu G Gindulyte A . PubChem substance and compound databases. Nucleic Acids Res. (2016) 44:D1202–1213. doi: 10.1093/nar/gkv951 26400175 PMC4702940

[63] Hou T Wang J Li Y Wang W . Assessing the performance of the MM/PBSA and MM/GBSA methods. 1. The accuracy of binding free energy calculations based on molecular dynamics simulations. J Chem Inf Model. (2011) 51:69–82. doi: 10.1021/ci100275a 21117705 PMC3029230

[64] Xie S Zhu H Huang N . AI-designed molecules in drug discovery, structural novelty evaluation, and implications. J Chem Inf Model. (2025) 65:8924–33. doi: 10.1021/acs.jcim.5c00921 40820734

[65] Rossen L Sirockin F Schneider N Grisoni F . Scaffold hopping with generative reinforcement learning. J Chem Inf Model. (2025) 65:6513–25. doi: 10.1021/acs.jcim.5c00029 40570167 PMC12264953

[66] Alakhdar A Poczos B Washburn N . Diffusion models in de novo drug design. J Chem Inf Model. (2024) 64:7238–56. doi: 10.1021/acs.jcim.4c01107 39322943 PMC11481093

[67] Zhang X Gao H Qi Y Li Y Wang R . Generation of rational drug-like molecular structures through a multiple-objective reinforcement learning framework. Molecules. (2024) 30:18. doi: 10.3390/molecules30010018 39795076 PMC11721775

[68] Boonpalit K Chuntakaruk H Kinchagawat J Wolschann P Hannongbua S Rungrotmongkol T . Pre-training strategy for antiviral drug screening with low-data graph neural network: A case study in HIV-1 K103N reverse transcriptase. J Comput Chem. (2025) 46:e27514. doi: 10.1002/jcc.27514 39434589

[69] Zalenski N Suo DJ Meredith BR Savoie DJ Naas MJ Betancourt D . Kinetic investigation of resistance to Islatravir conferred by mutations in HIV-1 reverse transcriptase. J Mol Biol. (2025) 437:169100. doi: 10.1016/j.jmb.2025.169100 40139311

[70] Cilento ME Kirby KA Sarafianos SG . Avoiding drug resistance in HIV reverse transcriptase. Chem Rev. (2021) 121:3271–96. doi: 10.1021/acs.chemrev.0c00967 33507067 PMC8149104

[71] Xavier Ruiz F Arnold E . Evolving understanding of HIV-1 reverse transcriptase structure, function, inhibition, and resistance. Curr Opin Struct Biol. (2020) 61:113–23. doi: 10.1016/j.sbi.2019.11.011 31935541 PMC7596924

[72] Ding J Das K Hsiou Y Sarafianos SG Clark AD Jr. Jacobo-Molina A . Structure and functional implications of the polymerase active site region in a complex of HIV-1 RT with a double-stranded DNA template-primer and an antibody Fab fragment at 2.8 A resolution. J Mol Biol. (1998) 284:1095–111. doi: 10.1006/jmbi.1998.2208 9837729

[73] Sarafianos SG Marchand B Das K Himmel DM Parniak MA Hughes SH . Structure and function of HIV-1 reverse transcriptase: Molecular mechanisms of polymerization and inhibition. J Mol Biol. (2009) 385:693–713. doi: 10.1016/j.jmb.2008.10.071 19022262 PMC2881421

[74] Zhang K Zhang YJ Li M Pannecouque C De Clercq E Wang S . Deciphering the enigmas of non-nucleoside reverse transcriptase inhibitors (NNRTIs): A medicinal chemistry expedition towards combating HIV drug resistance. Med Res Rev. (2025) 45:426–83. doi: 10.1002/med.22080 39188075

[75] Brüssow H . Recent developments in HIV antivirals: The prospect of prophylactic drugs to change the pandemic. Microb Biotechnol. (2026) 19:e70317. doi: 10.1111/1751-7915.70317 41693105 PMC12907452

[76] Seong JE Ahn SM Han M Lee YS Lee JA Ahn JY . Long-term effectiveness and tolerability of dolutegravir/lamivudine in Korea: A 3-year follow up study. Infect Chemother. (2026) 58:19–28. doi: 10.3947/ic.2025.0070 41943246 PMC13053887

[77] Sebata A Nagao T Matsukawa T Maeno Y Suzuki M Yoshikawa Y . Safety and effectiveness analysis of dolutegravir and dolutegravir/abacavir/lamivudine in Japanese people living with HIV: 10 years of post-marketing surveillance. Adv Ther. (2026). doi: 10.1007/s12325-026-03544-0 41910934 PMC13222196

[78] Rehman N Nguyen H . Nevirapine. In: Statpearls. StatPearls Publishing LLC, Treasure Island (FL (2024) 43:2527–49.

[79] Taki E Soleimani F Asadi A Ghahramanpour H Namvar A Heidary M . Cabotegravir/rilpivirine: The last FDA-approved drug to treat HIV. Expert Rev Anti Infect Ther. (2022) 20:1135–47. doi: 10.1080/14787210.2022.2081153 35596583

[80] Zhuang C Pannecouque C De Clercq E Chen F . Development of non-nucleoside reverse transcriptase inhibitors (NNRTIs): Our past twenty years. Acta Pharm Sin B. (2020) 10:961–78. doi: 10.1016/j.apsb.2019.11.010 32642405 PMC7332669

[81] Orkin C Kuritzkes DR Katlama C Su FH Plank RM Lahoulou R . Doravirine resistance patterns identified through week 192 in the DRIVE-FORWARD and DRIVE-AHEAD phase 3 clinical trials. J Acquir Immune Defic Syndr. (2026) 101:308–15. doi: 10.1097/qai.0000000000003787 41129125

[82] Sakakibara N Balboni G Congiu C Onnis V Demizu Y Misawa T . Design, synthesis, and anti-HIV-1 activity of 1-substituted 3-(3,5-dimethylbenzyl)triazine derivatives. Antivir Chem Chemother. (2015) 24:62–71. doi: 10.1177/2040206615612208 26514833 PMC5890519

[83] Su DS Lim JJ Tinney E Wan BL Young MB Anderson KD . Substituted tetrahydroquinolines as potent allosteric inhibitors of reverse transcriptase and its key mutants. Bioorg Med Chem Lett. (2009) 19:5119–23. doi: 10.1016/j.bmcl.2009.07.031 19631528

[84] Zhang J Zhan P Wu J Li Z Jiang Y Ge W . Synthesis and biological evaluation of novel 5-alkyl-2-arylthio-6-((3,4-dihydroquinolin-1(2H)-yl)methyl)pyrimidin-4(3H)-ones as potent non-nucleoside HIV-1 reverse transcriptase inhibitors. Bioorg Med Chem. (2011) 19:4366–74. doi: 10.1016/j.bmc.2011.05.024 21683601

[85] Chander S Wang P Ashok P Yang LM Zheng YT Murugesan S . Rational design, synthesis, anti-HIV-1 RT and antimicrobial activity of novel 3-(6-methoxy-3,4-dihydroquinolin-1(2H)-yl)-1-(piperazin-1-yl)propan-1-one derivatives. Bioorg Chem. (2016) 67:75–83. doi: 10.1016/j.bioorg.2016.05.009 27288643

[86] Chen S Zhou X Lu Y Xu K Wen J Cui M . Anti-HIV drugs lopinavir/ritonavir activate bitter taste receptors. Chem Senses. (2023) 48:1–9. doi: 10.1093/chemse/bjad035 37625013 PMC10486187

[87] Shalbi F Ali AR . A mini-review on integrase inhibitors: The cornerstone of next-generation HIV treatment. Eur J Med Chem. (2024) 279:116900. doi: 10.1016/j.ejmech.2024.116900 39332384

[88] Inciarte A Berrocal L Santos JR Aleman MR Curran A Medina Gonzalez R . Baseline resistance mutations and virological response to dolutegravir in treatment-naïve patients: A multicentre NGS study. J Antimicrob Chemother. (2026) 81:dkag080. doi: 10.1093/jac/dkag080 41873440

[89] Sax PE Arribas JR Orkin C Lazzarin A Pozniak A DeJesus E . Bictegravir/emtricitabine/tenofovir alafenamide as initial treatment for HIV-1: Five-year follow-up from two randomized trials. EClinicalMedicine. (2023) 59:101991. doi: 10.1016/j.eclinm.2023.101991 37200995 PMC10186485

[90] Tekeste SS Wilkinson TA Weiner EM Xu X Miller JT Le Grice SF . Interaction between reverse transcriptase and integrase is required for reverse transcription during HIV-1 replication. J Virol. (2015) 89:12058–69. doi: 10.1128/jvi.01471-15 26401032 PMC4645309

[91] Wang JY Ling H Yang W Craigie R . Structure of a two-domain fragment of HIV-1 integrase: Implications for domain organization in the intact protein. EMBO J. (2001) 20:7333–43. doi: 10.1093/emboj/20.24.7333 11743009 PMC125787

[92] Bonnard D Le Rouzic E Eiler S Amadori C Orlov I Bruneau JM . Structure-function analyses unravel distinct effects of allosteric inhibitors of HIV-1 integrase on viral maturation and integration. J Biol Chem. (2018) 293:6172–86. doi: 10.1074/jbc.M117.816793 29507092 PMC5912470

[93] Park JH Yun JH Shi Y Han J Li X Jin Z . Non-cryogenic structure and dynamics of HIV-1 integrase catalytic core domain by X-ray free-electron lasers. Int J Mol Sci. (2019) 20:1943. doi: 10.3390/ijms20081943 31010024 PMC6514806

[94] Bernatavicius A Šícho M Janssen APA Hassen AK Preuss M van Westen GJP . AlphaFold meets de novo drug design: Leveraging structural protein information in multitarget molecular generative models. J Chem Inf Model. (2024) 64:8113–22. doi: 10.1021/acs.jcim.4c00309 39475544 PMC11558674

[95] Li G Meanwell NA Krystal MR Langley DR Naidu BN Sivaprakasam P . Discovery and optimization of novel pyrazolopyrimidines as potent and orally bioavailable allosteric HIV-1 integrase inhibitors. J Med Chem. (2020) 63:2620–37. doi: 10.1021/acs.jmedchem.9b01681 32081010

[96] Patel M Naidu BN Dicker I Higley H Lin Z Terry B . Design, synthesis and SAR study of bridged tricyclic pyrimidinone carboxamides as HIV-1 integrase inhibitors. Bioorg Med Chem. (2020) 28:115541. doi: 10.1016/j.bmc.2020.115541 32389483

[97] Sharma H Sanchez TW Neamati N Detorio M SChinazi RF Cheng X . Synthesis, docking, and biological studies of phenanthrene β-diketo acids as novel HIV-1 integrase inhibitors. Bioorg Med Chem Lett. (2013) 23:6146–51. doi: 10.1016/j.bmcl.2013.09.009 24091080 PMC3866047

[98] Steigbigel RT Cooper DA Kumar PN Eron JE Schechter M Markowitz M . Raltegravir with optimized background therapy for resistant HIV-1 infection. N Engl J Med. (2008) 359:339–54. doi: 10.1056/NEJMoa0708975 18650512

[99] Walmsley SL Antela A Clumeck N Duiculescu D Eberhard A Gutiérrez F . Dolutegravir plus abacavir-lamivudine for the treatment of HIV-1 infection. N Engl J Med. (2013) 369:1807–18. doi: 10.1056/NEJMoa1215541 24195548

[100] Gallant J Lazzarin A Mills A Orkin C Podzamczer D Tebas P . Bictegravir, emtricitabine, and tenofovir alafenamide versus dolutegravir, abacavir, and lamivudine for initial treatment of HIV-1 infection (GS-US-380-1489): a double-blind, multicentre, phase 3, randomised controlled non-inferiority trial. Lancet. (2017) 390:2063–72. doi: 10.1016/s0140-6736(17)32299-7 28867497

[101] Orkin C Arasteh K Górgolas Hernández-Mora M Pokrovsky V Overton ET Girard PM . Long-acting cabotegravir and rilpivirine after oral induction for HIV-1 infection. N Engl J Med. (2020) 382:1124–35. doi: 10.1056/NEJMoa1909512 32130806

[102] Oliaie SS Safakish M Roudsari RV Mahboubi-Rabbani M Hajimahdi Z Zarghi A . Design, synthesis, docking studies, and biological evaluation of novel 2-hydroxyacetophenone derivatives as anti-HIV-1 agents. Curr HIV Res. (2023) 21:290–300. doi: 10.2174/011570162x261377231107110447 37990893

[103] Wang B He Y Wen X Xi Z . Prediction and molecular field view of drug resistance in HIV-1 protease mutants. Sci Rep. (2022) 12:2913. doi: 10.1038/s41598-022-07012-x 35190671 PMC8861105

[104] Rusconi S Paoletti N Supuran CT . HIV protease and its inhibition. Enzymes. (2025) 58:129–49. doi: 10.1016/bs.enz.2025.06.001 41238295

[105] Talha B Dhamoon AS . Ritonavir. In: Statpearls. StatPearls Publishing LLC, Treasure Island (FL (2024).

[106] Venkatachalam S Muralidharan N Pandian R Sayed Y Gromiha MM . Integrative computational approaches for understanding drug resistance in HIV-1 protease subtype C. Viruses. (2025) 17:850. doi: 10.3390/v17060850 40573441 PMC12197794

[107] Swain AL Miller MM Green J Rich DH Schneider J Kent SB . X-ray crystallographic structure of a complex between a synthetic protease of human immunodeficiency virus 1 and a substrate-based hydroxyethylamine inhibitor. Proc Natl Acad Sci USA. (1990) 87:8805–9. doi: 10.1073/pnas.87.22.8805 2247451 PMC55048

[108] Venkatachalam S Krishnan SR Sayed Y Gromiha MM . Structural and functional studies on HIV protease: mechanism of action, subtypes, inhibitors, and drug resistance. Methods Mol Biol. (2025) 2867:185–200. doi: 10.1007/978-1-0716-4196-5_11 39576582

[109] Sherry D Sheik Ismail Z Mokhantso T Sayed Y . Subtype-specific HIV-1 protease and the role of hinge and flap dynamics in drug resistance: a subtype C narrative. Viruses. (2025) 17:1044. doi: 10.3390/v17081044 40872759 PMC12390352

[110] Furui K Sakano K Ohue M . Predictive and therapeutic applications of protein language models. Allergol Int. (2025) 74:534–48. doi: 10.1016/j.alit.2025.08.004 40967982

[111] Zhou H Ma L Dong B Wang J Zhang G Wang M . Design, synthesis, and biological evaluation of novel HIV-1 protease inhibitors containing pyrrolidine-derived P2 ligands to combat drug-resistant variant. Eur J Med Chem. (2023) 255:115389. doi: 10.1016/j.ejmech.2023.115389 37120996

[112] Ngo B Sutton RE . Next-generation HIV-1 therapeutics in co-endemic settings. Biomedicines. (2026) 14:330. doi: 10.3390/biomedicines14020330 41751229 PMC12938469

[113] Fanjul F Gavalda M Campins A Ferré A Martín L Peñaranda M . HIV therapy: The latest developments in antiviral drugs-a scoping review. Biomedicines. (2025) 13:2629. doi: 10.3390/biomedicines13112629 41301723 PMC12650496

[114] Sayyed SK Quraishi M Rajasekharan SK Sonawane T Ravichandran V . Zinc C293 as a novel scaffold with dual activity against HIV-1 integrase and reverse transcriptase. Comput Biol Chem. (2025) 119:108573. doi: 10.1016/j.compbiolchem.2025.108573 40580592

[115] Soares TA Nunes-Alves A Mazzolari A Ruggiu F Wei GW Merz K . The (Re)-evolution of quantitative structure-activity relationship (QSAR) studies propelled by the surge of machine learning methods. J Chem Inf Model. (2022) 62:5317–20. doi: 10.1021/acs.jcim.2c01422 36437763

[116] Shahin R Jaafreh S Azzam Y . Tracking protein kinase targeting advances: integrating QSAR into machine learning for kinase-targeted drug discovery. Future Sci OA. (2025) 11:2483631. doi: 10.1080/20565623.2025.2483631 40181786 PMC11980485

[117] Burns JW Green WH . Generalizable, fast, and accurate DeepQSPR with fastprop. J Cheminform. (2025) 17:73. doi: 10.1186/s13321-025-01013-4 40361252 PMC12076996

[118] Manan A Baek E Ilyas S Lee D . Digital alchemy: The rise of machine and deep learning in small-molecule drug discovery. Int J Mol Sci. (2025) 26:6807. doi: 10.3390/ijms26146807 40725054 PMC12295739

[119] Behrooznia S Hooshmand M . A systematic review of drug-related interactions─utilizing deep learning and LLMs for prediction and mitigation. ACS Omega. (2025) 10:61048–75. doi: 10.1021/acsomega.5c04997 41476460 PMC12750216

